# Donafenib combined with sintilimab for advanced hepatocellular carcinoma: a single arm phase II trial

**DOI:** 10.1186/s12885-025-13605-2

**Published:** 2025-02-05

**Authors:** Xiaoyang Hong, Yongjian Guo, Wenbo Shi, Kangshun Zhu, Licong Liang, Liteng Lin, Ye Chen, Jingwen Zhou, Jingjun Huang, Jiabai Huang, Yaozhu Wu, Wensou Huang, Mingyue Cai

**Affiliations:** 1 Department of Minimally Invasive Interventional Radiology, the Second Affiliated Hospital, Guangzhou Medical University, Guangzhou, China; 2 Department of Radiology, Yuebei People’s Hospital, Shaoguan, China; 3 Shanghai Clinical Research Ward (SCRW), Shanghai Sixth People’s Hospital, Shanghai Jiao Tong University School of Medicine, Shanghai, China; 4 The Second School of Clinical Medicine, Guangzhou Medical University, Guangzhou, China

**Keywords:** Hepatocellular carcinoma, Tyrosine-kinase inhibitor, Donafenib, Immune-checkpoint inhibitor, Programmed death 1 inhibitor, Sintilimab, Combination therapy

## Abstract

**Background:**

Previous studies evaluating antiangiogenic agents plus immune checkpoint inhibitors for unresectable hepatocellular carcinoma (HCC) have shown encouraging results. This study was conducted to investigate the efficacy and safety of donafenib combined with sintilimab (Don-Sin) for advanced HCC.

**Methods:**

This was a single-center, single-arm phase II trial recruiting patients with BCLC stage C HCC. A safety run-in cohort was planned with the first 6 patients receiving oral donafenib 200 mg twice daily and intravenous sintilimab 200 mg once every 3 weeks. Dose-limiting toxicities (DLTs) were evaluated to determine the recommended dose of donafenib for those enrolled thereafter. The primary endpoint of this study was progression-free survival (PFS) per mRECIST.

**Results:**

30 patients were enrolled. As 3 patients (50.0%) experienced DLTs during safety run-in, the initial dose of donafenib was adjusted to 200 mg once daily for subsequent patients. The primary endpoint was met with a median PFS of 6.2 (95% confidence interval [CI], 4.4-8.0) months per mRECIST (6.3 [95% CI, 5.4–7.2] months per RECIST 1.1). The objective response rate was 23.3% per mRECIST and 16.7% per RECIST 1.1, while the disease control rate reached 76.7% per mRECIST/RECIST 1.1. The median overall survival was 16.0 (95% CI, 13.5–18.5) months. Treatment-related adverse events (TRAEs) occurred in 28 patients (93.3%) and grade 3 TRAEs were observed in 9 patients (30.0%).

**Conclusions:**

Don-Sin showed promising antitumor effects with an acceptable safety profile in patients with advanced stage HCC. The preliminary findings need to be further evaluated in phase III randomized controlled trials.

**Trial registration:**

ClinicalTrials.gov (identifier: NCT05162352; date of registration: December 4, 2021).

## Introduction

Hepatocellular carcinoma (HCC) is one of the deadliest cancers worldwide. Most cases are diagnosed at an advanced stage, often making them ineligible for curative treatments and resulting in a dismal prognosis [1–3].

Donafenib is a tyrosine-kinase inhibitor (TKI) derived from sorafenib [4]. Results of the phase II-III ZGDH3 trial demonstrated a superior overall survival (OS) of donafenib over sorafenib in Chinese patients with unresectable/metastatic HCC [5]. Thereafter, donafenib has been recommended as one of the first-line treatment options for advanced HCC in China [2, 6]. However, the efficacy of donafenib monotherapy is modest, with a median OS of only 12.1 months [5], necessitating the exploration of more effective treatments strategies to improve clinical outcomes of the patients.

In recent years, the application of immune checkpoint inhibitors (ICIs) has revolutionized the treatment of solid tumors, including HCC [7, 8]. Studies evaluating the combination of antiangiogenic agents and ICIs have shown encouraging results for advanced HCC [9–12]. In the phase III IMbrave150 trial, the combination of bevacizumab and atezolizumab provided a superior survival benefit over sorafenib in unresectable HCC patients [9]. Similarly, the phase II-III ORIENT-32 trial showed that a bevacizumab biosimilar plus sintilimab led to a longer OS versus sorafenib in Chinese patients with unresectable HCC [10]. Additionally, several trials revealed that lenvatinib combined with programmed death 1 (PD-1) inhibitors could provide remarkable survival benefits in unresectable/advanced HCC patients [11, 13, 14]. More recently, the phase III CARES-310 trial demonstrated longer progression-free survival (PFS) and OS with rivoceranib plus camrelizumab in advanced HCC patients when compared with sorafenib [12]. These results suggested that antiangiogenic agents combined with ICIs might be superior treatment strategies for advanced HCC.

Herein, we presented the findings of a single-arm phase II trial evaluating the efficacy and safety of donafenib combined with sintilimab (Don-Sin) as a first-line treatment for advanced stage HCC.

## Methods

### Study design and participants

This was a single-center, single-arm, open-label, phase II trial conducted at the Second Affiliated Hospital, Guangzhou Medical University with approval from the institutional ethics committee (2021-hs-68) and in accordance with the Declaration of Helsinki and Good Clinical Practice. All patients provided written informed consent. The study was registered at ClinicalTrial.gov (identifier: NCT05162352).

This study had a two-stage design, with a preliminary safety run-in cohort followed by an expansion cohort. In the safety run-in stage, 6 patients were enrolled and received concurrent oral donafenib (Suzhou Zelgen Biopharmaceuticals, Kunshan, China) 200 mg twice daily and intravenous sintilimab (Innovent Biologics, Suzhou, China) 200 mg once every 3 weeks. Tolerability of donafenib at this dose was assessed by dose-limiting toxicity (DLT) over the first 21 days after treatment initiation. If DLT events related to donafenib occurred in 2 or more patients, the initial dose of donafenib was reduced to 200 mg once daily for the subsequently enrolled patients. And the dose of donafenib would no longer be increased to 200 mg twice daily. The study treatment continued until disease progression, intolerable toxicity, withdrawal of informed consent, loss to follow-up, death, or other conditions requiring termination of the treatment, whichever occurred first. Sintilimab treatment lasted for up to 2 years. Treatment interruption, dose reduction (donafenib only) and treatment discontinuation due to toxicity was determined by the investigators according to the drug inserts. Patients were allowed to receive donafenib or sintilimab as a single treatment and were still considered to be under study if the other drug caused intolerable toxicity without disease progression.

In this study, patients with Barcelona Clinic Liver Cancer (BCLC) stage C HCC confirmed pathologically or clinically [2, 3] were evaluated. The main inclusion criteria were as follows: (1) age ≥ 18 years, (2) at least one measurable intrahepatic lesion based on modified Response Evaluation Criteria in Solid Tumors (mRECIST) and Response Evaluation Criteria in Solid Tumors version 1.1 (RECIST 1.1), (3) Eastern Cooperative Oncology Group performance status (ECOG PS) ≤1, (4) Child-Pugh score ≤7 with alanine aminotransferase and aspartate aminotransferase < 5 times upper limit of normal (ULN), total bilirubin ≤1.5×ULN, and albumin ≥ 28 g/L, (5) prothrombin time-international normalized ratio ≤1.5×ULN, (6) adequate hematologic function (leukocyte count ≥ 3.0 × 10^9^/L, neutrophil count ≥ 1.5 × 10^9^/L, platelet count ≥ 75 × 10^9^/L and hemoglobin ≥ 90 g/L), (7) creatinine ≤1.5×ULN, and (8) life expectancy ≥ 3 months. The main exclusion criteria were: (1) diffuse HCC, (2) tumor thrombus involving bilateral portal vein branches, main portal vein or vena cava, (3) central nervous system metastasis, (4) previous treatment with systemic therapy or hepatic arterial infusion chemotherapy, (5) uncontrolled hydrothorax or ascites, (6) variceal bleeding within 3 months before treatment initiation, (7) history of hepatic encephalopathy, (8) history of cell/organ transplantation, (9) history of human immunodeficiency virus infection, and (10) history of malignancy other than HCC.

### Assessments and endpoints

The assessment of tumor response was conducted by the investigators based on mRECIST and RECIST 1.1. Safety of the treatment was evaluated by monitoring adverse events (AEs) according to Common Terminology Criteria for Adverse Events version 5.0. In the safety run-in, DLT is defined as any ≥ grade 3 AE for non-haematological toxicity and any grade 4 AE for haematological toxicity [4].

The primary endpoint of this study was PFS per mRECIST. The secondary endpoints included PFS per RECIST 1.1, objective response rate (ORR), disease control rate (DCR), OS, and AEs. ORR was defined as the percentage of patients achieving complete response (CR) or partial response (PR). DCR was defined as the percentage of patients achieving CR, PR, or stable disease (SD). PFS was defined as the time from treatment initiation until disease progression or death, whichever occurred first. OS was defined as the time from treatment initiation until death from any reason.

### Follow-up

The follow-up ended on 31 August 2023. Baseline assessment was performed within 7 days before the treatment initiation. During the treatment, scheduled visit, hematological and biochemical tests were conducted every 3 weeks. Abdominal dynamic contrast-enhanced computed tomography or magnetic resonance imaging, and chest computed tomography were performed at least every 6 weeks. If clinically indicated, unscheduled assessments and other examinations could be carried out.

### Statistical analyses

Sample size was calculated by using PASS 14 software (NCSS, Kaysville, Utah, USA). Prior to the study, 14 patients with BCLC stage C HCC had been treated with donafenib at our institution with a median PFS of 3.4 months per mRECIST (data unpublished), which was similar to that (3.7 months per RECIST 1.1) for patients with unresectable/metastatic HCC treated with donafenib in the ZGDH3 trial [5]. It was hypothesized that the addition of sintilimab to donafenib could improve the median PFS by 2.5 months to 5.9 months. To detect the difference in PFS with a one-sided alpha error of 5%, a power of 80%, and a recruitment/follow-up period of 9 months, 27 patients were required. Considering a 10% dropout, a sample size of 30 patients was planned.

Statistical analyses were performed using SPSS Statistics 26 (IBM, Armonk, New York, USA). Continuous variables were presented as means ± standard deviations or medians with ranges, as appropriate. Categorical variables were presented as frequencies (percentages). 95% confidence intervals (CIs) for ORR and DCR were calculated using the Clopper-Pearson method. PFS and OS were estimated by the Kaplan-Meier method.

## Results

### Patients and treatment

**Fig. 1:**
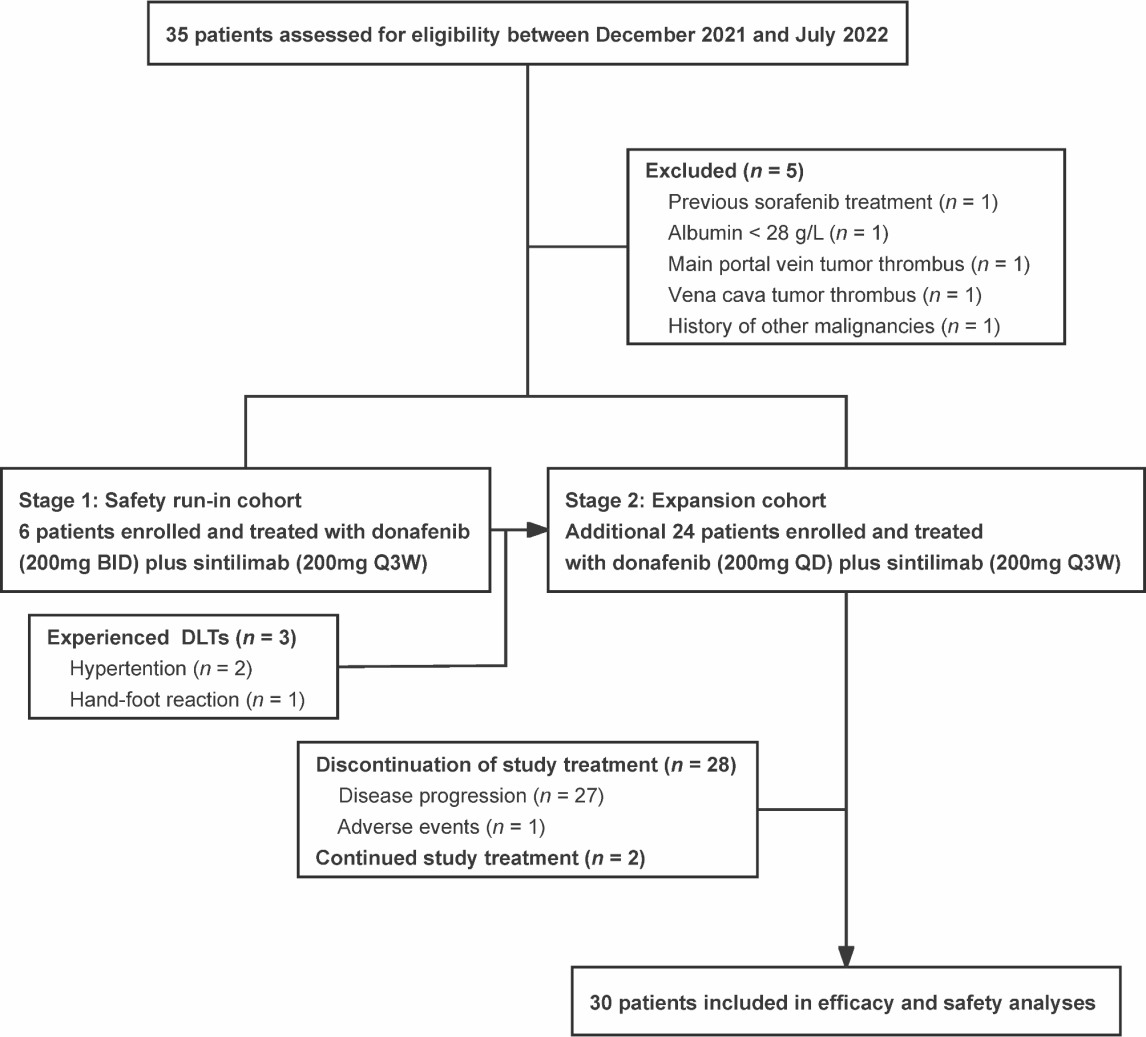
Trial flow diagram. BID, twice daily; Q3W, once every 3 weeks; QD, once daily; DLT, dose-limiting toxicity

Between 11 December 2021 and 18 January 2022, 35 patients were evaluated for eligibility, and 30 of them were enrolled (Fig. 1). In the safety run-in stage, 3 of the 6 patients (50.0%) experienced DLTs. Therefore, the initial dose of donafenib was adjusted to 200 mg once daily for the subsequent patients enrolled.

The baseline characteristics of the patients were summarized in Table 1. Twenty-four patients (80.0%) had previously received local treatments, of whom 4 (13.3%) had undergone hepatectomy, 3 (10.0%) had undergone thermal ablation, and 19 (63.3%) had undergone transarterial chemoembolization. The mean largest tumor diameter was 8.9 ± 3.8 (range, 3.2–14.8) cm, with 16 patients (53.3%) each having intrahepatic tumor number > 3 and a bilobar tumor distribution. Twenty-four patients (80.0%) presented with macrovascular invasion and 19 patients (63.3%) presented with extrahepatic metastases.

**Table 1:** Baseline characteristics of the patients

Characteristic	Value
Age (years)	
Mean ± SD	56.3 ± 10.4
≥ 60/<60, *n* (%)	11 (36.7)/19 (63.3)
Sex, *n* (%)	
Female/Male	3 (13.3)/27 (86.7)
ECOG PS, *n* (%)	
1/0	8 (26.7)/22 (73.3)
Cause of HCC	
Hepatitis B^a^	28 (93.3)
Hepatitis C^b^	1 (3.3)
Alcohol intake^c^	1 (3.3)
HBV-DNA (IU/mL)^d^	
≥ 1000/<1000, *n* (%)	12 (42.9)/16 (57.1)
Child-Pugh class, *n* (%)	
A/B	26 (86.7)/4 (13.3)
ALBI grade, *n* (%)	
1/2	9 (30.0)/21 (70.0)
α-Fetoprotein (µg/L)	
Median (range)	412.20 (49.82-285142.0)
≥ 200/<200, *n* (%)	22 (73.3)/8 (26.7)
PIVKA-II (mAU/mL)	
Median (range)	671.31 (25.0-198274.0)
≥ 400/<400, *n* (%)	19 (63.3)/11 (36.7)
Largest tumor diameter (cm)	
Mean ± SD	8.9 ± 3.8
≥ 10/<10, *n* (%)	13 (43.3) /17 (56.7)
Number of intrahepatic tumors, *n* (%)	
> 3/≤3	16 (53.3)/14 (46.7)
Tumor distribution, *n* (%)	
Bilobar/Unilobar	16 (53.3)/14 (46.7)
Macrovascular invasion, *n* (%)	24 (80.0)
Extrahepatic metastasis, *n* (%)	19 (63.3)
Lune	15 (50.0)
Lymph nodes	7 (23.3)
Bone	2 (6.7)
Adrenal gland	1 (3.3)
Previous local treatment, *n* (%)	24 (80.0)
Hepatectomy	4 (13.3)
Thermal ablation	3 (10.0)
Transarterial chemoembolization	19 (63.3)

HCC, hepatocellular carcinoma; SD, standard deviation; ECOG PS, Eastern Cooperative Oncology Group performance status; HBV, hepatitis B virus; PIVKA-II, protein induced by vitamin K absence or antagonist-II; ALBI, albumin-bilirubin

^a^Twenty-eight patients had hepatitis B-related cirrhosis, and all received antiviral therapy with entecavir, tenofovir disoproxil fumarate or tenofovir alafenamide during study treatment

^b^This patient had a history of hepatitis C-related cirrhosis but was negative for hepatitis C virus-RNA

^c^The patient had alcoholic cirrhosis but had abstained from alcohol before enrollment

^d^Two patients without hepatitis B virus infection were excluded

**Fig. 2:**
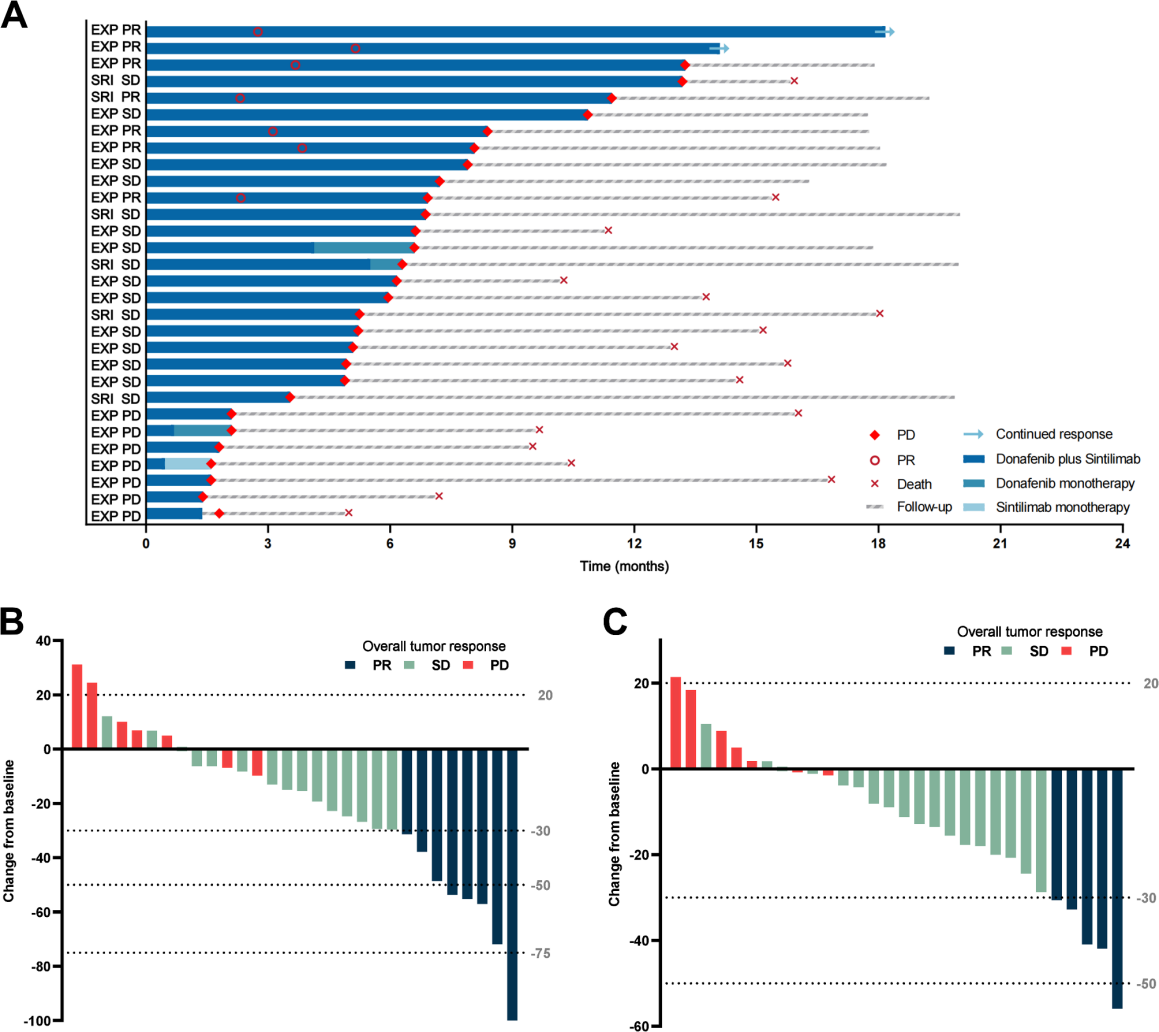
Antitumor activity. Duration of treatment and response assessments by mRECIST (**A**). Best percentage change from baseline in diameters of intrahepatic target lesions per mRECIST (**B**) and RECIST 1.1 (**C**). EXP, expansion cohort; SRI, safety run-in cohort; PR, partial response; SD, stable disease; PD, progressive disease; mRECIST, modified Response Evaluation Criteria in Solid Tumors; RECIST 1.1, Response Evaluation Criteria in Solid Tumors version 1.1

The duration of treatment and follow-up in individuals was shown in Fig. 2A. As of the cut-off date (31 August 2023), 2 patients were still on treatment. The median follow-up for the patients was 16.0 (range, 5.0-20.1) months. The mean duration of donafenib administration was 6.6 ± 4.3 (range, 1.4–18.2) months, and the mean treatment cycles of sintilimab was 9.1 ± 6.2 (range, 1–26).

After the termination of study treatment, 28 patients (93.3%) underwent post-study treatment. Among them, 10 (33.3%) still received Don-Sin and 18 (60.0%) received other systemic therapies. Additionally, 19 patients (63.3%) underwent additional local-regional treatment (Table 2).

**Table 2:** Post-study treatments for the patients

Post-study treatment	Number of patients
Continued Don-Sin	10
Other TKIs	18
Lenvatinib	9
Regorafenib	8
Apatinib	1
Other PD-1 inhibitors	10
Tislelizumab	9
Camrelizumab	1
Transarterial chemoembolization	13
Hepatic arterial infusion chemotherapy	6
Iodine-125 seed brachytherapy	5

Don-Sin, donafenib plus sintilimab; TKI, tyrosine-kinase inhibitor; PD-1, programmed death 1

### Efficacy

The efficacy outcomes were detailed in Table 3. The ORR and DCR were 23.3% (95% CI, 9.9-42.3%) and 76.7% (95% CI, 57.7-90.1%), respectively, per mRECIST, and 16.7% (95% CI, 5.6-34.7%) and 76.7% (95% CI, 57.7-90.1%), respectively, per RECIST 1.1. None of the patients achieved CR per mRECIST/RECIST 1.1. Shrinkage of the target lesions was observed in 23 patients (76.7%) per mRECIST/RECIST 1.1 (Fig. 2B, C).

**Table 3:** Efficacy outcomes for the patients

Outcomes	mRECIST	RECIST 1.1
Tumor response		
CR, *n* (%)	-	-
PR, *n* (%)	7 (23.3)	5 (16.7)
SD, *n* (%)	16 (53.3)	18 (60.0)
PD, *n* (%)	7 (23.3)	7(23.3)
ORR, % (95% CI)	23.3 (9.9–42.3)	16.7 (5.6–34.7)
DCR, % (95% CI)	76.7 (57.7–90.1)	76.7 (57.7–90.1)
Median PFS, months (95% CI)	6.2 (4.4-8.0)	6.3 (5.4–7.2)
Median OS, months (95% CI)	16.0 (13.5–18.5)	

mRECIST, modified Response Evaluation Criteria in Solid Tumors; RECIST 1.1, Response Evaluation Criteria in Solid Tumors version 1.1; CR, complete response; PR, partial response; SD, stable disease; PD, progressive disease; CI, confidence interval; ORR, objective response rate; DCR, disease control rate; PFS, progression-free survival; OS, overall survival

**Fig. 3:**
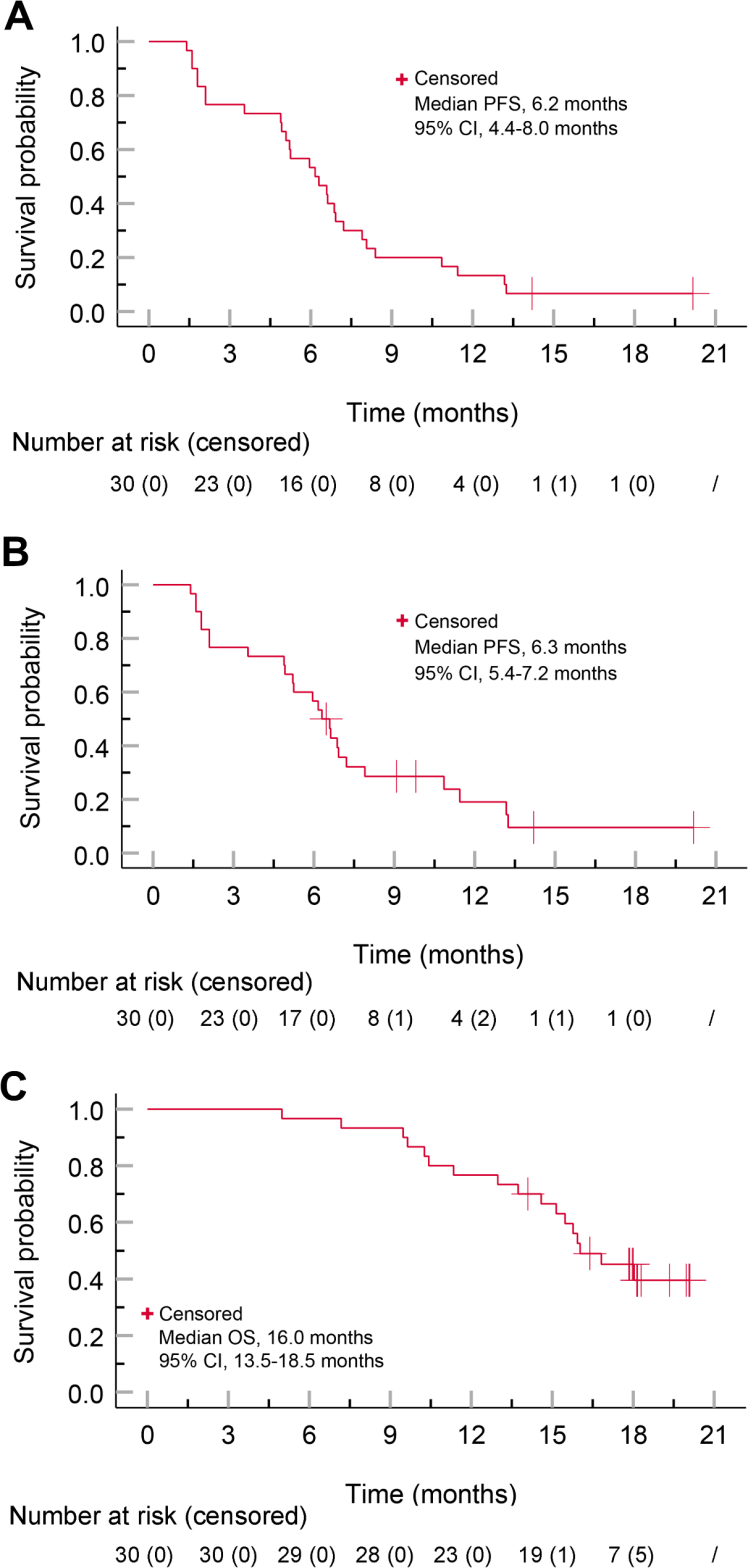
Kaplan-Meier analyses of progression-free survival and overall survival. Analyses of progression-free survival per mRECIST (**A**) and RECIST 1.1 (**B**). Analysis of overall survival (**C**). PFS, progression-free survival; OS, overall survival; CI, confidence interval; mRECIST, modified Response Evaluation Criteria in Solid Tumors; RECIST 1.1, Response Evaluation Criteria in Solid Tumors version 1.1

During the study period, 28 patients (93.3%) experienced disease progression. The median PFS was 6.2 (95% CI, 4.4-8.0) months per mRECIST (Fig. 3A) and 6.3 (95% CI, 5.4–7.2) months per RECIST 1.1 (Fig. 3B). By the time of data analysis, 17 patients (56.7%) had died. The causes of death included tumor progression in 12 patients (70.6%), variceal hemorrhage in three (17.6%), liver failure in one (5.9%), and pneumonia and sepsis in one (5.9%). The median OS was 16.0 (95% CI, 13.5–18.5) months (Fig. 3C).

### Safety

The safety run-in cohort consisted of six patients, three of whom experienced DLTs, including grade 3 hypertension in two and grade 3 hand-foot reaction in one. In the total cohort, treatment-related AEs (TRAEs) were observed in 28 (93.3%) patients (Table 4). The most prevalent TRAEs included hand-foot reaction (36.7%), decreased platelet (30.0%), increased aspartate aminotransferase (30.0%), diarrhea (20.0%), rash (20.0%), decreased lymphocyte (20.0%), hypothyroidism (20.0%), and proteinuria (20.0%). Grade 3 TRAEs occurred in 9 patients (30.0%), with hypertension being the most frequent grade 3 TRAE (6.7%), while no grade 4/5 TAREs were observed.

**Table 4:** Treatment-related adverse events

Adverse events	Any grade	Grade 1/2	Grade 3
Total, *n* (%)	28 (93.3)	28 (93.3)	9 (30.0)
Hand-foot reaction	11 (36.7)	10 (33.3)	1 (3.3)
Decreased platelet	9 (30.0)	8 (26.7)	1 (3.3)
Increased AST	9 (30.0)	9 (30.0)	-
Diarrhea	6 (20.0)	5 (16.7)	1 (3.3)
Rash	6 (20.0)	5 (16.7)	1 (3.3)
Decreased lymphocyte	6 (20.0)	5 (16.7)	1 (3.3)
Hypothyroidism	6 (20.0)	6 (20.0)	-
Proteinuria	6 (20.0)	6 (20.0)	-
Hypertension	5 (16.7)	3 (10.0)	2 (6.7)
Alopecia	5 (16.7)	5 (16.7)	-
Decreased white blood cell	5 (16.7)	5 (16.7)	-
Anemia	4 (13.3)	3 (10.0)	1 (3.3)
Decreased weight	4 (13.3)	4 (13.3)	-
Increased ALT	4 (13.3)	4 (13.3)	-
Increased GGT	4 (13.3)	4 (13.3)	-
Fatigue	4 (13.3)	4 (13.3)	-
Increased TBil	4 (13.3)	4 (13.3)	-
Nausea	4 (13.3)	4 (13.3)	-
Elevated uric acid	4 (13.3)	4 (13.3)	-
Hypophosphatemia	3 (10.0)	3 (10.0)	-
Increased ALP	3 (10.0)	3 (10.0)	-
Hypoalbuminemia	3 (10.0)	3 (10.0)	-
Ventosity	3 (10.0)	3 (10.0)	-
Insomnia	3 (10.0)	3 (10.0)	-
Infusion reaction	3 (10.0)	3 (10.0)	-
Hyperthyroidism	2 (6.7)	2 (6.7)	-
Stomatitis/Gingival bleeding	2 (6.7)	2 (6.7)	-
Vomiting	2 (6.7)	2 (6.7)	-
Anorexia	2 (6.7)	2 (6.7)	-
Hyponatremia	2 (6.7)	2 (6.7)	-
Pneumonitis	1 (3.3)	-	1 (3.3)
Arthralgia	1 (3.3)	1 (3.3)	-
Hypokalemia	1 (3.3)	1 (3.3)	-

AST, aspartate aminotransferase; ALP, alkaline phosphatase; ALT, alanine aminotransferase; TBil, total bilirubin; GGT, γ-glutamyl transpeptidase

TRAEs led to interruption, dose reduction and discontinuation of donafenib in 6 (20.0%), 4 (13.3%) and 2 (6.7%) patients, respectively, and interruption and discontinuation of sintilimab in 7 (23.3%) and 4 (13.3%) patients, respectively. Discontinuation of both drugs occurred in only one patient (3.3%).

## Discussion

This phase II trial evaluated Don-Sin in the treatment of advanced stage HCC and met the prespecified primary endpoint with a median PFS of 6.2 months per mRECIST (6.3 months per RECIST 1.1). Our results showed that the combination therapy had promising antitumor activity against advanced HCC, with favourable tumor response and survival outcomes, as well as a manageable safety profile, indicating that Don-Sin is a viable therapeutic option for advanced stage HCC.

In the ZGDH3 trial [5], the median PFS of donafenib for unresectable/metastatic HCC was 3.7 months per RECIST 1.1. Similarly, in 14 patients with advanced stage HCC who had previously received donafenib at our institution, the median PFS was 3.4 months per mRECIST (data unpublished). In the present study, with the addition of sintilimab to donafenib, the median PFS per mRECIST was extended by 2.8 months. Additionally, this combination achieved an ORR of 23.3% (per mRECIST; 16.7% per RECIST 1.1), a DCR of 76.7% (per mRECIST/RECIST 1.1), and a median OS of 16.0 months. These results were superior to those of donafenib monotherapy in the ZGDH3 trial [5]. It was notable that the patients enrolled in our study had a higher tumor burden and more advanced disease (all patients had BCLC stage C disease, 80.0% had previously received local treatments, and 53.3% had intrahepatic tumor number > 3/bilateral tumor distribution, with a mean largest tumor diameter of 8.9 cm), which undoubtedly led to poor tumor control and prognosis [2, 3, 15, 16]. Even so, Don-Sin still achieved promising therapeutic outcomes in these patients, suggesting that the combination therapy might be a more effective treatment for advanced HCC.

Currently, several ICIs are available with certain therapeutic benefit in advanced HCC [7, 17]. Although there was still a lack of sufficient data for sintilimab monotherapy in the treatment of HCC [18], its combination therapy has been confirmed to have significant therapeutic effects on advanced disease [10, 19–23]. The ORIENT-32 trial is a phase II-III study evaluating sintilimab plus a bevacizumab biosimilar for unresectable HCC, which showed that this combination yielded a higher ORR and longer PFS and OS compared to sorafenib [10]. In addition, previous studies [19–23], including ours, have also demonstrated the feasibility and effectiveness of sintilimab combination therapy. The results of the present study once again supported these findings.

Previous studies have shown that giving HCC patients a reduced initial dose of sorafenib compared to a full dose may not compromise the survival [24, 25]. In this study, the initial dose of donafenib was adjusted to 200 mg once daily, as DLTs occurred in 3 patients during safety run-in. From the tumor response and survival results of our study, it seemed that reducing the initial dose of donafenib did not attenuate the efficacy in tumor control, and its combination with sintilimab achieved positive clinical outcomes in the advanced HCC patients.

At present, the exact mechanisms that lead to the treatment benefits of Don-Sin are still not well understood. Some preclinical studies uncovered that low-dose sorafenib could promote antitumor immunity by modulating multiple immune cells and the tumor microenvironment, suggesting the potential for sorafenib to be combined with immunotherapy [26–28]. As donafenib is a derivative of sorafenib with similar antitumor mechanisms [4, 5], it might also have similar antitumor immunomodulatory activities. In our study, the patients enrolled just received a reduced dose of donafenib. We therefore surmised that the combination of low-dose donafenib and sintilimab might have synergistic antitumor effects, thus contributing to the favourable results in the patients.

In this study, no unexpected AEs attributable to the combination therapy were observed. All TRAEs were consistent with previously reported AEs for each treatment [5, 29]. During the study, 28 patients (93.3%) experienced at least one any grade TRAE and 9 (30.0%) experienced grade 3 TRAEs, but none suffered from grade 4/5 TRAEs. As a result of the reduced initial dose of donafenib, the incidence of donafenib-related AEs (e.g., hypertension and hand-foot reaction) was significantly decreased [5]. These results suggested an acceptable safety and tolerability profile of the combination therapy of Don-Sin.

Additionally, although some patients with hepatitis B virus (HBV) infection and high HBV-DNA load (≥ 1000 IU/mL) were enrolled in this study, no hepatitis flares occurred with the use of sintilimab. This finding was consistent with those of our previous studies [19, 20] and suggested that PD-1 inhibitors (e.g. sintilimab) may be safe for patients with high baseline HBV-DNA if they are able to receive antiviral therapy while on anti-PD-1 therapy.

There were several limitations in our study. Firstly, it was a single-arm phase II trial without a control group for comparison. Secondly, the sample size of this study was small. These might limit the interpretation of the outcomes of the combination therapy. In addition, the open-label nature of the study might affect the assessments of efficacy and safety by the investigators. Therefore, the findings of this study should be considered as preliminary and need to be confirmed by large sample size, randomized controlled trials.

## Conclusions

In conclusion, this phase II study revealed that Don-Sin was a potential therapeutic strategy for patients with advanced stage HCC. The combination therapy showed promising clinical outcomes with favourable tumor response, PFS and OS, as well as an acceptable safety profile.

## Data Availability

The datasets used and/or analysed during the current study are available from the corresponding author on reasonable request.

## Abbreviations

AE: Adverse event
BCLC: Barcelona Clinic Liver Cancer
CI: Confidence interval
CR: Complete response
DCR: Disease control rate
DLT: Dose-limiting toxicity
Don-Sin: Donafenib plus sintilimab
ECOG PS: Eastern Cooperative Oncology Group performance status
HBV: Hepatitis B virus
HCC: Hepatocellular carcinoma
ICI: Immune-checkpoint inhibitor
mRECIST: Modified Response Evaluation Criteria in Solid Tumors
ORR: Objective response rate
OS: Overall survival
PD-1: Programmed death 1
PFS: Progression-free survival
PR: Partial response
RECIST 1.1: Response Evaluation Criteria in Solid Tumors version 1.1
SD: Stable disease
TKI: Tyrosine-kinase inhibitor
TRAE: Treatment-related adverse event
ULN: Upper limit of normal
